# Enhanced Platelet-Rich Plasma (ePRP) Stimulates Wound Healing through Effects on Metabolic Reprogramming in Fibroblasts

**DOI:** 10.3390/ijms222312623

**Published:** 2021-11-23

**Authors:** Hsin-Pei Weng, Yuan-Yang Cheng, Hsin-Lun Lee, Tai-Yi Hsu, Yu-Tang Chang, Yao-An Shen

**Affiliations:** 1 ICare Stem Cell Research Center, Taipei 100, Taiwan; bell.weng@icareyou.com.tw; 2 Department of Physical Medicine and Rehabilitation, Taichung Veterans General Hospital, Taichung 40705, Taiwan; rifampin@gmail.com; 3 Department of Radiation Oncology, Taipei Medical University Hospital, Taipei 110301, Taiwan; b001089024@tmu.edu.tw; 4 Department of Radiology, School of Medicine, College of Medicine, Taipei Medical University, Taipei 110301, Taiwan; 5 Taipei Cancer Center, Taipei Medical University, Taipei 110301, Taiwan; 6 Department of Pathology, School of Medicine, College of Medicine, Taipei Medical University, Taipei 110301, Taiwan; b101109024@tmu.edu.tw (T.-Y.H.); b101108034@tmu.edu.tw (Y.-T.C.); 7 Graduate Institute of Clinical Medicine, College of Medicine, Taipei Medical University, Taipei 110301, Taiwan; 8 International Master/Ph.D. Program in Medicine, College of Medicine, Taipei Medical University, Taipei 110301, Taiwan

**Keywords:** platelet-rich plasma, regenerative medicine, ageing, metabolic reprogramming, glycolysis

## Abstract

As a source of growth factors for expediting wound healing and tissue regeneration, plasma-rich plasma (PRP) has been extensively applied in diverse fields including orthopaedics, ophthalmology, oral and maxillofacial surgery, dentistry, and gynaecology. However, the function of PRP in metabolic regulations remains enigmatic. A standardized method was devised herein to enrich growth factors and to lyophilize it as enhanced PRP (ePRP) powder, which could become ubiquitously available without mechanical centrifugation in clinical practice. To identify metabolic reprogramming in human dermal fibroblasts under ePRP treatment, putative metabolic targets were identified by transcriptome profiling and validated for their metabolic effects and mechanism. ePRP does not only promote wound healing but re-aligns energy metabolism by shifting to glycolysis through stimulation of glycolytic enzyme activity in fibroblasts. On the contrary, oxygen consumption rates and several mitochondrial respiration activities were attenuated in ePRP-treated fibroblasts. Furthermore, ePRP treatment drives the mitochondrial resetting by hindering the mitochondrial biogenesis-related genes and results in a dampened mitochondrial mass. Antioxidant production was further increased by ePRP treatment to prevent reactive oxygen species formation. Besides, ePRP also halts the senescence progression of fibroblasts by activating *SIRT1* expression. Importantly, the glycolytic inhibitor 2-DG can completely reverse the ePRP-enhanced wound healing capacity, whereas the mitochondrial inhibitor oligomycin cannot. This is the first study to utilize PRP for comprehensively investigating its effects on the metabolic reprogramming of fibroblasts. These findings indicate that PRP’s primary metabolic regulation is to promote metabolic reprogramming toward glycolytic energy metabolism in fibroblasts, preserving redox equilibrium and allowing anabolic pathways necessary for the healing and anti-ageing process.

## 1. Introduction

Geriatric health is becoming increasingly vital as people live longer lives and the number and proportion of elderly people rise sharply in most developed and developing countries. The cardiovascular system, skeletal system, digestive system, bladder and urinary tract, teeth, and eyes are some of the biological systems or physiological functions that will deteriorate as we age. When it comes to geriatric issues, the topics that currently attract extensive attention are ways to sustain healthy skin and delay skin ageing. Skin ageing is a complicated biological process that can be divided into two categories as chronological (intrinsic) ageing, which is driven mostly by genetic factors, and photoageing (extrinsic), which is caused by solar UV exposure [1,2]. The chronological ageing of human skin is characterized by increasing laxity, fine wrinkling, and dermal atrophy, as well as diminished quantities of type I and III fibrillar collagens [2,3]. Photoageing is characterized by dry skin, dampened skin elasticity, fragmentation of collagen and elastic fibres, epidermal atrophy, and even solar elastosis, an accumulation of aberrant elastotic material with an accentuated loss in type I and III collagens, and the presence of dermal infiltrates [2,4].

Platelet-rich plasma (PRP) therapy is recently receiving rapidly growing attention and becoming a focus of research in orthopaedics, dermatology, sports-related injuries, and cancer biology [5]. PRP is a non-surgical, minimally-invasive therapy that provides a tailored delivery system for concentrated growth factors and biomolecules required for spurring tissue repair, making it excellent for anti-ageing and regenerative medicine [6]. Treatments using PRP can revitalize the skin [7]. Various “split-face” studies (patients served as their own control, injecting PRP randomized to either the left or right side of their face) are undertaken to illustrate the benefit of PRP for rejuvenation [8]. It is particularly worth mentioning that the combination of PRP and hyaluronic acid resulted in a notable improvement in skin condition [9]. To prevent skin ageing, the mechanisms of PRP that facilitate skin repair and regeneration have been explored. By stimulating the synthesis of matrix metalloproteinases (MMPs), PRP can not only accelerate cutaneous fibroblast growth but also eliminate photodamaged extracellular matrix (ECM) components [10]. It also boosts the production of ECM components including type I collagen and elastin [11]. From a histological perspective, there is a clinical trial that shows PRP’s potential to rejuvenate photoaged facial skin [12].

Platelet concentrates are exceptional endogenous therapeutic agents that have inspired interest in a wide range of medical and dental fields due to their immense potential to aid soft and hard tissue regeneration [13]. The efficacy of platelet concentrates and PRP in oral and maxillofacial surgery has been extensively researched during the last two decades [14]. Autologous platelet concentrates may be beneficial in the prevention and treatment of medication-related osteonecrosis of the jaw attributed to their local immunomodulatory properties and the ability of platelet factors to induce angiogenesis and tissue regeneration [15]. Similarly, platelet-rich fibrin was shown to be equally effective as triamcinolone acetonide in limiting the severity and symptomatology of oral lichen planus lesions [16]. Advanced platelet-rich fibrin and leukocyte-platelet-rich fibrin are vastly superior to typical hemostatics in strengthening clot stabilization, minimizing post-surgical bleeding, and accelerating wound healing in dental extraction [17]. Nevertheless, preparations of platelet concentrates and PRP vary in cell and platelet composition, making consistent research and therapeutic application problematic. Employing lyophilized PRP, clinicians may be able to apply a specific quantity of growth factors by using a defined amount of PRP powder. Furthermore, PRP powder might become readily accessible as a dry substance that does not require centrifugation and is ideal for long-term storage, improving efficiency in clinical practice. Consequently, before implementing the findings of this fundamental scientific study into clinical practice, a standardized PRP preparation process must be certified. We have established a standardized technique for refining and activating platelets to generate lyophilized enhanced PRP (ePRP) powder. Having a primary understanding of the physiological impact of ageing on the skin, along with PRP’s potent skin rejuvenation and repair capacity, we would like to take a step further and investigate the role of PRP in mitochondrial metabolism, oxidative stress, and ageing-related cellular mechanisms in skin fibroblasts.

Our cells are exposed to reactive oxygen species (ROS) as we age, which results in mitochondrial malfunction and redox imbalance [18]. ROS are produced by the skin’s redox imbalance and exogenous physical insults, like UV exposure [19]. Several types of oxidative damage, such as lipid peroxidation [20], protein oxidation [21], inflammasome activation, and senescence, are triggered by mitochondrial malfunction and the accumulation of oxidative stress. Mitochondrial dysfunction and oxidative stress are crucial aspects of a multitude of skin disorders [22] and cancers [23]. Researchers recently discovered that PRP-combined therapy can spur skin wound healing and reduce local inflammation, which helps to counteract oxidative stress [24,25]. Since both inflammation and redox homeostasis are linked to cell metabolism, tissue repair, and ageing, we can postulate that ePRP may have a direct impact on the link between mechanical events and underlying energy metabolism. Despite the fact that PRP treatments have been utilized for a variety of indications for over three decades, the metabolic regulation of PRP and the molecular mechanisms driving the metabolic reprogramming of targeted cells remain elusive. To resolve these questions, we employed human dermal fibroblasts as a model to delve into the characteristics of ePRP-driven metabolic reprogramming.

## 2. Results

### 2.1. ePRP Stimulates Wound Healing by Releasing Critical Growth Factors

**Figure 1:**
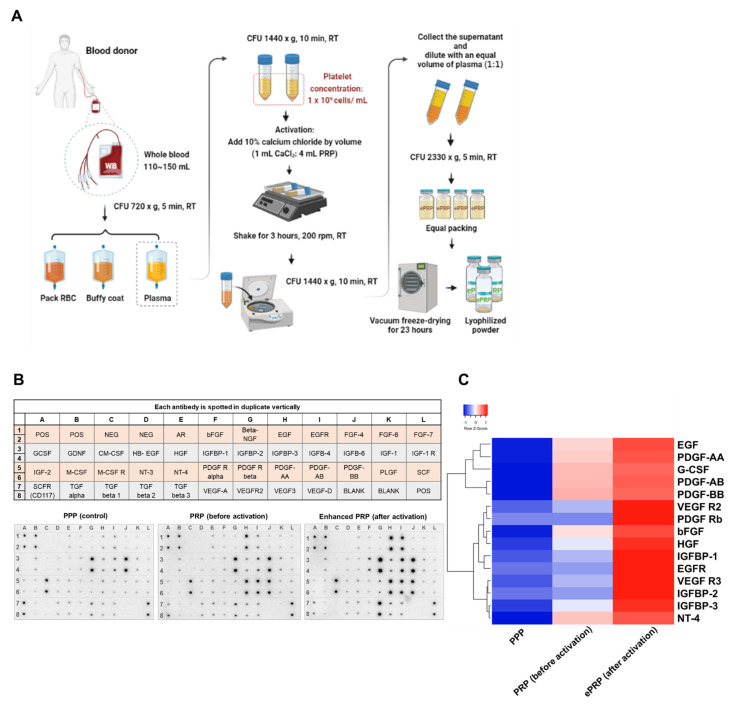
Characterization of enhanced platelet-rich plasma (ePRP). (**A**) The flowchart of ePRP preparation (**B**) Human growth factor cytokine array map. Representative dot plots from 40 different types of cytokine array analyses for PPP, PRP (before activation), and ePRP (after activation). (**C**) Heatmap of chemokine and cytokine protein levels in PPP, PRP (before activation), and ePRP (after activation) in (**B**). The relative expression levels of the proteins according to a clustering behaviour.

We started by establishing a standardized approach to provide enhanced platelet-rich plasma (ePRP) through designated centrifugation, activation, and lyophilization processes because the preparations and ingredients of PRP injections can vary massively (Figure 1A). After blood bag centrifugation, we separated 110–150 mL whole blood from a healthy donor into three layers: plasma, buffy coat (white-coloured layer containing white blood cells and platelets), and erythrocyte (pack red blood cells). PRP with a concentration of 1 × 10^9^ cells per mL was generated by centrifuging plasma. All PRP samples were activated for 3 h at 200 rpm shaking speed with a 10% calcium chloride (CaCl_2_) solution in a 1/4 volume of total PRP solution. Finally, all water moisture was drained by vacuum freeze-drying through lyophilization to yield enhanced PRP (ePRP) powder.

**Figure 2:**
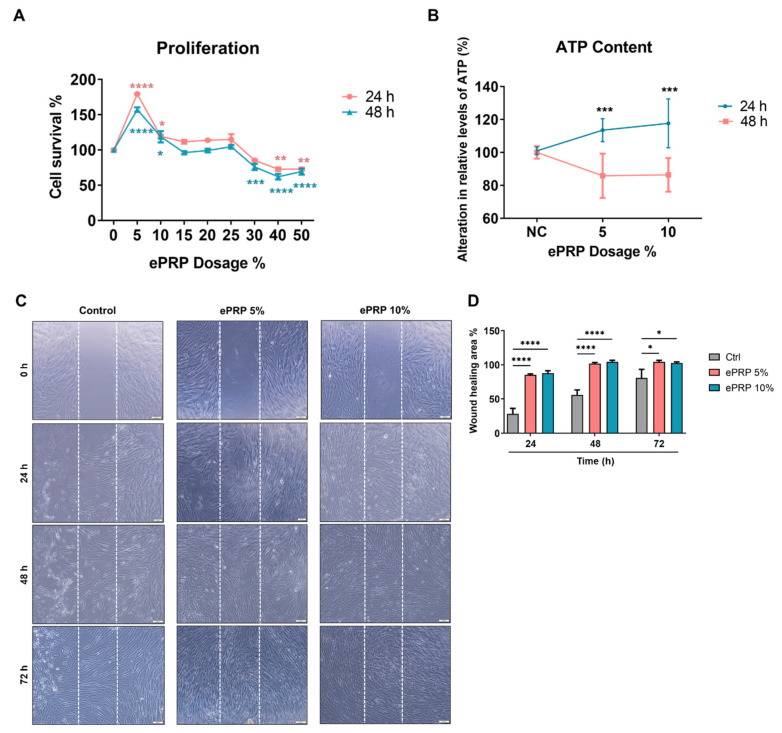
ePRP promotes wound healing ability in fibroblasts. (**A**) Fibroblasts treated for 24 and 48 h with ePRP. The relative viability rate was determined using the CCK-8 test, and all treatment groups were compared to the vehicle control group (100%). The data is shown as mean ± SEM (N = 2, N denotes the number of independent experiments performed). (**B**) Fibroblast ATP levels after treatment with 5% and 10% ePRP for 24 and 48 h. At 24 h, there was a considerable rise in ATP, which was dosage-dependent. The data are shown as mean ± SEM (N = 2). (**C**) Wound healing migration experiments were performed in fibroblasts at 0, 24, 48, and 72 h in 5% and 10% ePRP pre-treated cells, with untreated cells serving as a control. The area covered by the cells at 0, 24, 48, and 72 h after wounding is depicted in illustrative microscope images. The scale bars indicate a distance of 100 μm. (**D**) The statistical outcomes of migration area percentage of independently repeated experiments as mean ± SEM (N = 3) were shown in bar graphs. The two-way ANOVA with Sidak’s multiple comparisons test is used to evaluate all ePRP treatments statistically (* *p* < 0.05, ** *p* < 0.01, *** *p* < 0.001, **** *p* < 0.0001).

By using PRP without activation and platelet-poor plasma (PPP) as references through a cytokine array assay, we determined that epidermal growth factor (EGF), epidermal growth factor receptor (EGFR), basic fibroblast growth factor (bFGF), hepatocyte growth factor (HGF), insulin-like growth factor-binding protein (IGFBP), platelet-derived growth factor receptors (PDGF-R), and vascular endothelial growth factor receptors (VEGFR) were substantially concentrated in ePRP (Figure 1B,C). The proliferation rate of fibroblasts was significantly increased when treated with ePRP powder at concentrations of 5% and 10% but declined when the concentration of ePRP was greater than 30% (Figure 2A). In fibroblasts, ePRP concentrations of 5% and 10% promoted ATP generation (Figure 2B). The migration of fibroblasts with 5% and 10% ePRP was improved greatly in the wound-healing experiment, with an 80–90% wound closure after 24 h (Figure 2C). These findings demonstrated that the ePRP contains enriched growth factors that help fibroblasts proliferate and repair wounds.

### 2.2. ePRP Triggers a Metabolic Reprogramming in Fibroblasts

**Figure 3:**
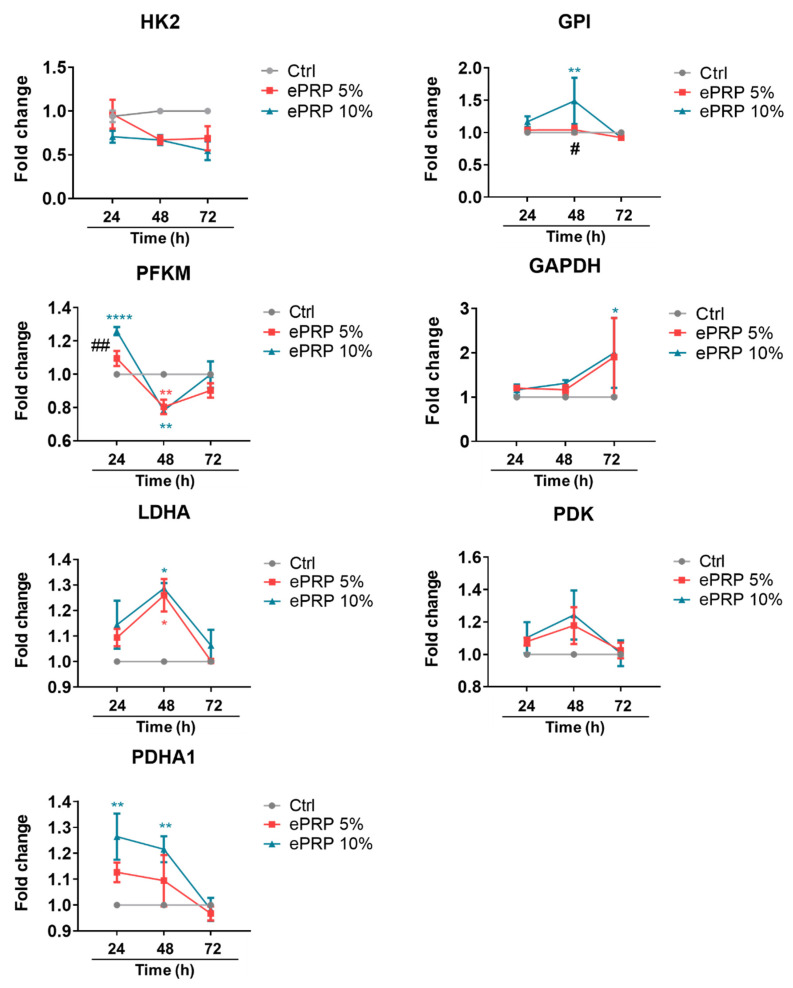
The gene expression of glycolytic enzymes in fibroblasts is stimulated by ePRP. Quantitative real-time PCR was utilized to evaluate the mRNA expression levels of multiple glycolytic enzymes in fibroblasts treated with ePRP for 24, 48, and 72 h. The 2^−ΔΔCt^ method was used to calculate relative gene expression of fold changes, employing EEF1A1 as the internal control. Two-way ANOVA with Tukey’s multiple comparisons test was used to determine statistical significance. * illustrates the statistical difference between each group (* *p* < 0.05, ** *p* < 0.01, **** *p* < 0.0001); ^#^ shows the statistical difference between 24 and 48 h (^#^ *p* < 0.05, ^##^ *p* < 0.01). The data are shown as mean ± SEM (N = 5).

Most glycolytic enzymes were considerably altered at different time points after ePRP treatment, according to the kinetics of the expression pattern representing glycolysis activity (Figure 3). After 24 h, PFKM and PDHA1 were elevated, whereas GPI and LDHA were up-regulated at 48 h (Figure 3). GAPDH was gradually activated and showed a sharp rise in gene expression at 72 h, whereas most ePRP-stimulated glycolytic enzymes exhibited a similar trend in gene expression at 72 h as that of the control (Figure 3). These results revealed that ePRP dynamically activate several glycolytic enzymes to modulate and sustain glucose metabolism to meet energy demands in different periods.

**Figure 4:**
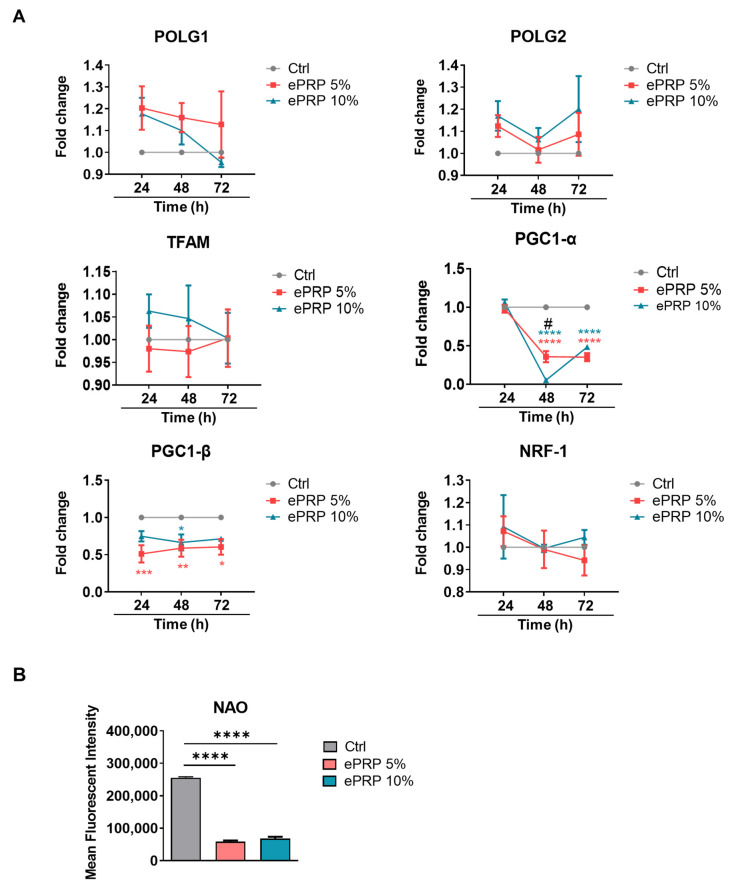
ePRP causes mitochondrial resetting by down-regulating mitochondrial biogenesis-related genes. (**A**) The line graph depicted the fold changes in mRNA expression levels of mitochondrial biogenesis-related genes in fibroblasts. Ct values were determined using quantitative real-time PCR for 24, 48, and 72 h after ePRP pretreatment. Two-way ANOVA with Tukey’s multiple comparisons test was used to determine statistical significance (* *p* < 0.05, ** *p* < 0.01, *** *p* < 0.001, **** *p <* 0.0001). * illustrates the statistical difference between each group; # shows the statistical difference between 24 and 48 h. The data are shown as mean ± SEM (N = 5). (**B**) Flow cytometry analysis for mitochondrial mass measurement. A representative bar chart of mitochondria stained with NAO is shown under indicated treatments. The results of an ordinary one-way ANOVA with **** *p* < 0.0001 and means ± SEM were shown (N = 2).

PGC-1α, PGC-1β, and PRC are transcriptional coactivators of the PGC-1 family, which play a pivotal role in the regulation of mitochondrial biogenesis and respiratory function [26]. ePRP restricts mitochondrial biogenesis and aerobic respiration by downregulating the expression of PGC-1 family members (Figure 4A), indicating that ePRP may repress mitochondrial biogenesis and aerobic respiration. Nonyl acridine orange (NAO) demonstrated that ePRP therapy diminished the mitochondrial mass in fibroblasts, as envisaged (Figure 4B).

**Figure 5:**
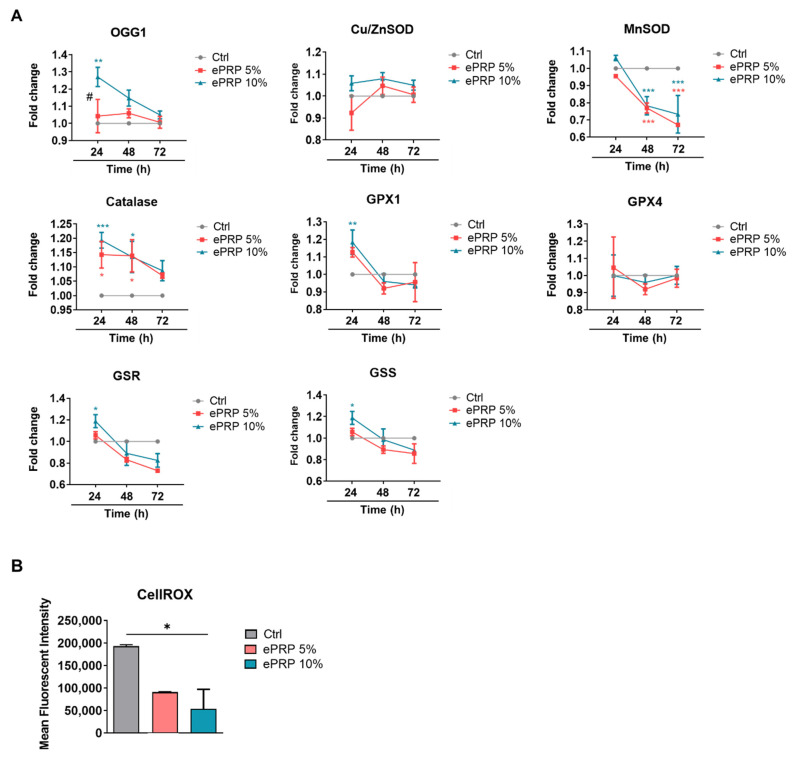
ePRP elevates antioxidant activity and reduces oxidative stress. (**A**) The relative mRNA level of antioxidant enzymes was measured in the absence of PRP or after pretreatment with 5% and 10% ePRP for 24 h, using EEF1A1 as an internal control gene. The experimental groups were expressed relative to the control group (baseline). The data are shown as mean ± SEM (N = 5). Two-way ANOVA with Tukey’s multiple comparisons test was used to verify statistical significance (* *p* < 0.05, ** *p <* 0.01, *** *p* < 0.001). * illustrates the statistical difference between each group; # shows the statistical difference between 24 and 48 h. (**B**) CellROX green was used to identify intracellular ROS following ePRP therapy and vehicle control. The ROS levels of mean fluorescence intensity (MFI) in untreated and ePRP-treated fibroblasts were compared using an ordinary one-way ANOVA, and the results were shown as means ± SEM (N = 2, * *p* < 0.05).

Following 24 h of ePRP administration in fibroblasts, multiple antioxidant enzymes, such as 8-oxoguanine DNA glycosylase-1 (OGG1), catalase (CAT), glutathione peroxidase 1 (GPX1), glutathione reductase (GSR), and glutathione synthetase (GSS) are up-regulated (Figure 5A). Manganese superoxide dismutase (MnSOD), on the other hand, was dramatically down-regulated at later periods (48 h and 72 h), most likely due to the current activation of other antioxidants (Figure 5A). Nonetheless, the augmented antioxidant defence system dramatically eliminates intracellular ROS (Figure 5B).

### 2.3. ePRP Impedes the Mitochondrial Activities in Fibroblasts

**Figure 6:**
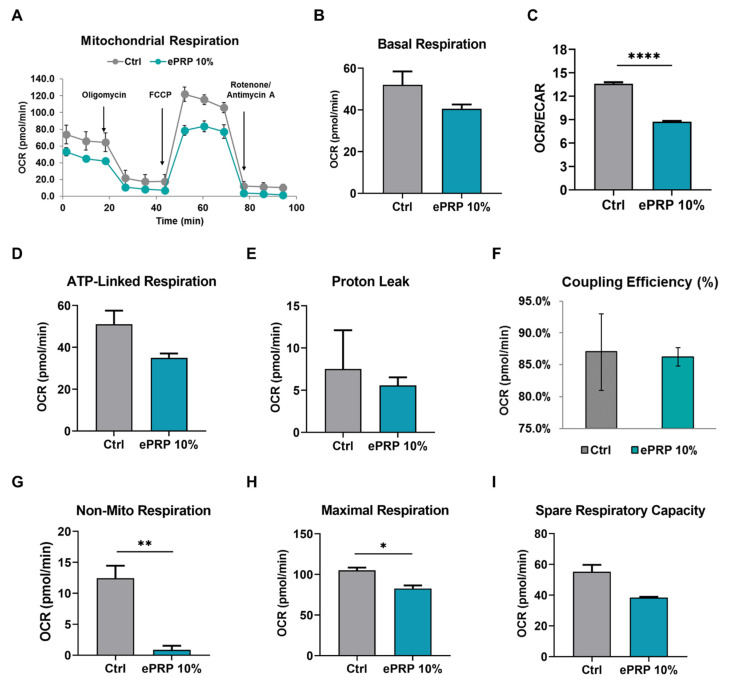
Mitochondrial function in fibroblasts is altered after exposure to ePRP. (**A**) All cells were treated with oligomycin, FCCP, and rotenone/antimycin A to quantify OCR and ECAR. A representative Seahorse Cell Mito Stress Test analysis with computable parameters. Values for the following respiratory parameters were determined. (**B**) Basal respiration. (**C**) OCR/ECAR. (**D**) ATP-linked respiration. (**E**) Proton leak. (**F**) Coupling efficiency (%) (**G**) Non-mitochondrial respiration. (**H**) Maximal respiration. (**I**) Mitochondrial spare respiratory capacity. * *p* < 0.05, ** *p* < 0.01, **** *p* < 0.0001 depicts that the groups differ significantly.

The oxygen consumption rate (OCR) and extracellular acidification (ECAR) were evaluated before and after the addition of inhibitors to obtain different mitochondrial parameters to scrutinize the impact of ePRP on mitochondrial functioning in fibroblasts (Figure 6A). ePRP stifled basal respiration (with a reduction trend, but it did not reach statistical significance) (Figure 6B) and the OCR/ECAR ratio (with a very significant reduction) (Figure 6C) in fibroblasts, indicating that the ePRP-driven metabolic shift depends on glycolysis rather than mitochondrial oxidative phosphorylation (OXPHOS) for energy generation. The lack of sensitivity to oligomycin in ePRP-treated fibroblasts is likely attributed to the low activity of ATP synthase, which is required to provide the ATP demand by mitochondria, and hence the effect of oligomycin is modest (Figure 6D).

The proportion of basal mitochondrial OCR used for ATP synthesis is used to compute coupling efficiency, which is the proportion of oxygen used to drive ATP synthesis compared to that inciting proton leak (ATP-linked OCR/basal OCR) [27]. The oxidation of substrates is coupled to the phosphorylation of ADP to ATP throughout OXPHOS. Mitochondria pump protons out to develop a protonmotive force, which is subsequently utilized to propel protons back via the ATP synthase, leading to the production of ATP. Some protons, unfortunately, leak back across the membrane, lessening the coupling efficiency. We disclosed that proton leak (or ion motions requiring proton motive force) showed a lessened trend in fibroblasts administered with ePRP (Figure 6E), and coupling efficiency is retained (Figure 6F). It is worth noting that non-mitochondrial oxygen consumption in fibroblasts before and after ePRP application is markedly different (Figure 6G). In ePRP-treated fibroblasts, reduced non-mitochondrial respiration (Figure 6G) may be associated with a lower ROS accumulation (Figure 5B).

The discrepancy between basal and maximal respiratory capacity is regarded as mitochondrial spare respiratory capacity (Figure 6H,I). Once cells are agitated by stress, their energy requirement escalates, requiring more ATP to maintain biological activities. Fibroblasts without ePRP pretreatment may need to generate more ATP to withstand stress, including oxidative stress, so they require a stronger spare and maximal respiratory capacity (Figure 6I) [28]. On the contrary, maximal respiratory capacity was significantly attenuated following ePRP therapy (Figure 6H) because it can up-regulate antioxidants to cope with oxidative stress.

### 2.4. SIRT1 Activation by ePRP Halts Senescence-Associated Features in Fibroblasts

**Figure 7:**
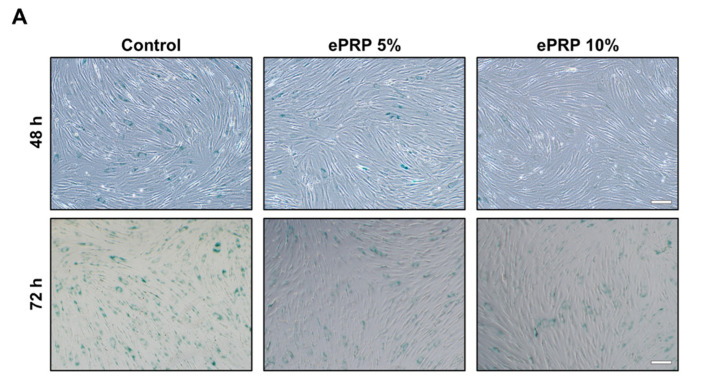

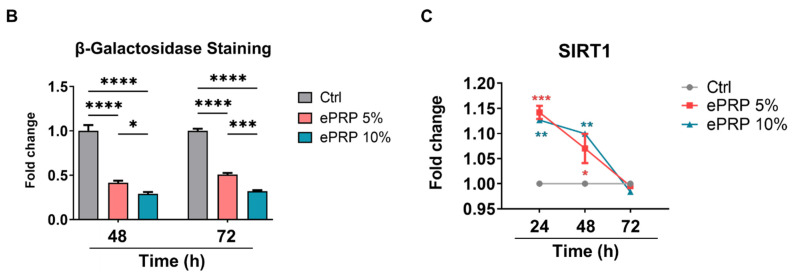
ePRP delays the ageing process in fibroblasts. (**A**) Cells with or without ePRP administration were fixed and stained for senescence β-galactosidase for 48 and 72 h. Microscope images depict senescence β-galactosidase activity intensity in each group. The scale bars indicate a distance of 100 μm. (**B**) ImageJ software was employed to quantify the fold change of cells positive for senescence β-galactosidase activity. The fold changes are shown as the means ± SEM of three independent experiments (* *p* < 0.05, *** *p* < 0.001, **** *p* < 0.0001). (**C**) The level of *SIRT1* mRNA expression was up-regulated in fibroblasts treated with ePRP, as shown by the line chart. Quantitative qPCR was used to determine the gene expression at 24, 48, and 72 h after ePRP treatment. Two-way ANOVA with Tukey’s multiple comparisons test was used to determine statistical significance (* *p* < 0.05, ** *p* < 0.01, *** *p* < 0.001) and means ± SEM were shown (N = 3).

The accumulation of excessive amounts of ROS has been connected to the rate of ageing [29]. Senescent cells generate more ROS, which is largely discharged from defective mitochondria [30]. The emergence of pH-dependent β-gal, the development of senescence-associated heterochromatin foci (SAHF), the persisting DNA damage response (DDR), and the senescence-associated secretory phenotype (SASP) have all been phenotypic characteristics of the senescent cells [31]. This transition in cell state is typically associated with increases in senescence-associated-galactosidase (SA-β-gal) activity, rendering any substrate for this enzyme a viable biomarker for distinguishing senescent cells when labelled specifically [32]. In a dose-dependent fashion, we observed that ePRP can significantly block SA-β-gal activity (Figure 7A,B).

The down-regulation of SIRT1 could be related to cellular senescence [33]. Nuclear SIRT1 is recognized as an autophagy substrate in senescence and is amenable to cytoplasmic autophagosome-lysosome degradation through the autophagy protein LC3 [34]. Restoration of SIRT1 might be a crucial way to reprogram aged cells, alluding to a possible therapeutic intervention to defer the ageing/senescence process [34]. As evidenced by dampened SA-β-gal staining, the overexpression of *SIRT1* (Figure 7C) by ePRP resulted in delayed senescence.

### 2.5. Glycolytic Inhibition Abolishes the ePRP-Stimulated Wound Healing Capacity of Fibroblasts

**Figure 8:**
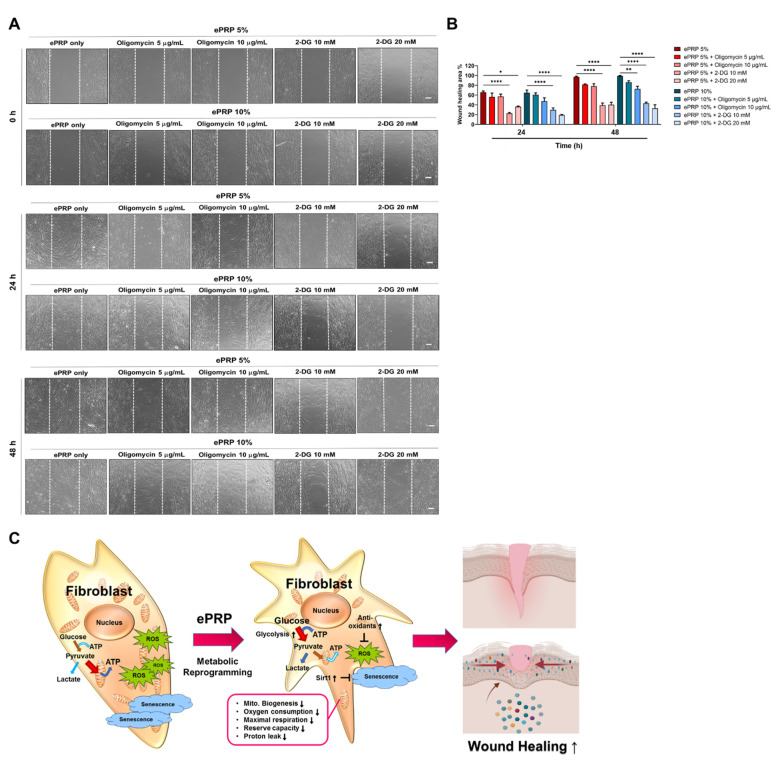
The glycolytic inhibitor 2-DG substantially reverses the ePRP-stimulated wound-healing ability. (**A**) After pre-treating cells with 5% and 10% ePRP for 24 h, the glycolytic inhibitor 2-DG and mitochondrial inhibitor oligomycin were applied for 48 h. Pictures of wound-healing migration were taken at 0, 24, and 48 h until full gap closure. The scale bars indicate a distance of 100 μm. (**B**) The statistical outcomes of migration area percentage of independently repeated experiments as mean ± SEM (N = 3) were shown in bar graphs. The two-way ANOVA with Sidak’s multiple comparisons test is used to evaluate all ePRP treatments statistically (* *p* < 0.05, ** *p* < 0.01, **** *p* < 0.0001). (**C**) The central metabolic control of ePRP in fibroblasts is depicted in this diagram. In fibroblasts, ePRP steers metabolic reprogramming toward glycolytic energy metabolism, ensuring redox balance and enabling anabolic pathways necessary for healing and anti-ageing.

In ePRP-treated fibroblasts, our findings revealed a rewiring of cellular metabolism with a shift toward glycolysis. These results warrant further investigation to determine whether ePRP-simulated glycolytic shift can serve as a companion metabolic adjustment or an absolute prerequisite for wound healing which will most likely be reversed by glucose inhibition. 2-DG acts as a d-glucose mimic, blocking glycolysis through the intracellular aggregation of 2-deoxy-d-glucose-6-phosphate (2-DG6P), disrupting the functionality of hexokinase and glucose-6-phosphate isomerase [35], and entirely reverting the ePRP-stimulated wound healing ability (Figure 8A,B). On the other hand, oligomycin, a prominent mitochondrial inhibitor, only partially affected the wound healing potential conferred by ePRP (Figure 8A,B). The results demonstrated that the switch to glycolysis is an absolute prerequisite for ePRP-stimulated wound healing (Figure 8C).

## 3. Discussion

PRP’s biological activities imitate and affect biological processes including anti-inflammation, analgesia, angiogenesis, and wound healing, making it a prospective therapeutic option. A recent academic debate centres on the feasibility of platelet concentrates used as a surgical adjuvant to enhance wound healing and accelerate tissue regeneration [15,16,17,36]. Autologous platelet concentrates have been employed for regenerative medicine, and they appear to aid soft-tissue wound healing by supplying greater quantities of autologous growth factors than natural concentrations [37]. PRP activation consists of (1) platelet degranulation to release growth factors, bioactive proteins from α-granules that promote cell mitosis, angiogenesis, chondrogenesis, and chemotaxis, and (2) fibrinogen cleavage to initiate matrix formation, a clotting process that allows the formation of a platelet gel and thus confines molecule secretion to the wound site [38]. Some clinicians inject PRP in its resting state, depending on the platelet activation that occurs naturally following exposure to the connective tissue’s endogenous collagen [39]. Platelets can also be activated by CaCl_2_, thrombin, or collagen type I to launch the healing process in injured tissues [40]. CaCl_2_ activation delivers much more growth factors than collagen activation and a less dense fibrin matrix than thrombin activation, promoting platelet entrapment and cell migration at the injection site [40]. Furthermore, CaCl_2_ activation can lead to a progressive release of growth factor over time, with a lower initial level followed by a continually growing amount of growth factor released [40]. As such, we employed a 3-h CaCl_2_ incubation to activate platelets and the release of critical growth factors including EGF, EGFR, bFGF, HGF, IGFBP, PDGF-R, and VEGFR was vastly elevated following CaCl_2_ activation compared to non-activated PRP (Figure 1B,C). EGF, bFGF, HGF, PDGF and VEGF can stimulate glucose metabolism [41,42,43,44,45]. Albeit that fibroblasts produce most of their energy through mitochondrial respiration, ePRP activates glycolytic enzymes, triggering a metabolic shift to aerobic glycolysis. The glycolytic shift is in line with the attenuated mitochondrial function and this manifests as low OCR/ECAR, maximal respiratory capacity, and non-mitochondrial respiration (e.g., low ROS generation) (Figure 6D,G,I).

Although glycolysis releases fewer ATP molecules per unit of glucose, it is a much quicker process than oxidative phosphorylation (OXPHOS), therefore cells will prioritize glycolysis during times of stress, such as after an injury [46]. The augmented ATP production of ePRP-treated fibroblasts depicted that the glycolytic flux was high enough to retain the energy status of the glycolytic shift (Figure 2B). Lactate, a glycolysis byproduct, is shown to be raised in the wound site shortly after wounding and to be elevated much above those in blood, demonstrating that the high lactate environment may drive collagen production in fibroblasts [47]. In recent work using single-cell resolution and the spatiotemporal observation of MDCKII cells, the leading edge of the advancing cell layer is shown to become progressively more migratory and to predominately utilize glycolysis for energy production when compared to the unwounded epithelial layer, which is non-migratory [48,49,50,51]. This metabolic shift implies that the migratory layer has a higher metabolic demand, which is supplied by glycolytic energy metabolism, which is fast but inefficient compared with OXPHOS [51]. In another study, zebrafish with their tails chopped off have a metabolic change that favours glucose metabolism during early regeneration [52]. Blocking glucose metabolism with 2-DG did not affect the embryo’s general health, but it prevented the tail from regrowing after amputation due to the inability to develop a functioning blastema, reinforcing that glycolysis plays a quintessential role in wound healing [52]. In our study, abrogating the energy production by 2-DG drastically reduced ePRP-mediated wound healing abilities in fibroblasts (Figure 8A,B). These clues are pieced up to decipher how ePRP directs the metabolic reprogramming of fibroblasts to cope with endogenous rapid energy consumption and exogenous redox stress for the benefit of the wound-healing process.

In terms of mitochondrial biogenesis, nucleus-encoded TFAM and mitochondrial DNA polymerase gamma (POLG), as well as its accessory component POLG2, is required for mtDNA transcription [53]. PGC-1 family members PGC-1α and PGC-1β and NRF1 modulate mitochondrial gene expression. PGC-1α also interacts with NRF1 to activate TFAM and increase NRF1 expression, resulting in positive feedback loops [54,55]. In fibroblasts with ePRP treatment, mitochondrial biogenesis-related genes such as PGC-1α and PGC-1β were dramatically suppressed, resulting in a reduction in mitochondrial mass (Figure 4A,B). Reduced mitochondrial biogenesis may coincide with the glycolytic shift of fibroblasts after ePRP therapy to dampen ROS production from the mitochondria.

The fundamental aspect of redox imbalance is increased intracellular ROS, which plays a pivotal role in skin ageing and damage processes. The buildup of ROS damages DNA, triggers an inflammatory response in the skin, disrupts the expressions of antioxidant enzymes, impedes collagen production by activating the nuclear factor kappa B (NF-κB) and activator protein 1 (AP–1), and intensifies MMPs, which decompose collagen and binding proteins in the dermis, culminating in skin ageing [56,57,58,59]. To maintain the oxidative equilibrium, the skin utilizes antioxidants, such as ascorbic acid, tocopherols, CAT, SOD, and GPX [60]. Intriguingly, our findings disclosed that ePRP can up-regulate the expression of antioxidants in fibroblasts, including OGG1, CAT, GPX1, GSR, and GSS (Figure 5A). Because electron leak from the mitochondrial respiratory chain is the primary source of intracellular ROS, preference for glycolysis in ePRP-treated fibroblasts may shield them from excessive ROS generation (Figure 5B). Additionally, glycolysis may potentially boost antioxidant defences by supplying NADPH via glucose 6-phosphate dehydrogenase (G6PD), which is then used to replenish reduced glutathione via glutathione reductase. Besides, fibroblast cybrids with altered mitochondria and greater quantities of mitochondrial ROS are less efficacious at closing wounds than cells with lower levels of mitochondrial ROS, signifying that mitochondrial ROS are essential and beneficial mediators for efficient wound healing [61]. In agreement with these findings, ePRP-driven augmented antioxidant defence systems leading to low ROS levels may confer anti-ageing and healing advantages to the fibroblasts with more effective wound closure.

The formation of senescent cells is a canonical feature of aged tissues [62]. SIRT1 has been implicated in the regulation of a variety of cellular and physiological processes, including ageing and metabolism [63,64,65]. SIRT1 deacetylates its substrates, which include histone substrates including acetylated histones H4K16 and H3K56, as well as non-histone targets such as p53 [66]. Through the engagement of ERK and S6K1 signalling, SIRT1 accelerated cell proliferation and counteracted cellular senescence in human diploid fibroblasts [67]. Surprisingly, we discovered that ePRP can promote the transcription of SIRT1 in fibroblasts, hence delaying cellular senescence (Figure 7C).

Increasing evidence supports the importance of metabolic reprogramming in tissue regeneration. By in-depth study, we found that glycolytic metabolism is quintessential for fibroblasts to heal the wound. In a metabolic shift, ePRP-treated fibroblasts showed a greater dependence on glycolysis compared with the control group. ePRP stimulates glucose metabolism to a level that is in excess of that needed to simply support cellular demand during the phases of wound healing. On the other hand, ePRP reactivates antioxidants to counteract the redox stress and senescence, which helps fibroblasts that have fisted antioxidant defence systems during wound healing and ageing. As PRP has been investigated to be applied in many fields, governing the metabolic reprogramming by PRP for healing is particularly noteworthy and awaits further investigation.

## 4. Materials and Methods

### 4.1. Preparation of Enhanced Platelet-Rich Plasma (ePRP)

Healthy participants provided 110–150 mL of whole blood in a blood bag containing CPDA-1 (JMS, Hiroshima City, Japan). The study was conducted according to the guidelines of the Declaration of Helsinki, and approved by the Institutional Review Board of Taichung Veterans General Hospital (protocol code: CF12251B-8). The concentration of platelets in 1 mL of whole blood was determined using automated blood analysis. Five-minute 3-layer centrifugation of the whole blood at 720× *g* delivered plasma, buffy coat, and pack RBC. Plasma was subsequently centrifuged for 10 min at 1440× *g* to obtain PRP with a concentration of 1 × 10^9^ cells per mL. Then, 10% calcium chloride was added by 1/4 volume of the total PRP solution and shaken at 200 rpm for 3 h to activate. After centrifuging the activated PRP for 10 min at 1440× *g*, the supernatant was diluted 1:1 with platelet-poor plasma (PPP). It was centrifuged at 2330× *g* for 5 min, and an equal aliquot was deposited in sterile injection vials. Subsequently, all water moisture was drained by vacuum freeze-drying to generate enhanced PRP (ePRP) powder by lyophilization.

### 4.2. Human Growth Factor Cytokine Array

Platelet poor plasma (PPP) and ePRP were assayed for the presence of human growth factors using a cytokine array (Cat. AAH-GF-1, RayBiotech, Peachtree Corners, GA, USA) and performed according to the manufacturer’s instructions. The data analysis has been employed by RayBiotech Microsoft^®^ Excel-based Analysis Software Tools for automated analysis (N = 40 individual human growth factor cytokines were detected).

### 4.3. Cell Culture

Human skin fibroblast cell line CCD-966SK was purchased from Bioresource Collection and Research Center, BCRC (Hsinchu, Taiwan). The cell line was cultured in Minimum Essential Medium (Cat. MT-10-010-CV, Corning, Manassas, VA, USA) containing 10% fetal bovine serum (Cat. 10437028, Gibco™ Thermo Fisher Scientific, Waltham, MA, USA) and supplemented with 1% antibiotic-antimycotic (Cat. MT-30-004-CI, Corning, NY, USA), 0.1 mM non-essential amino acids (MT-25-025-CI, Corning), 1.5 g/L sodium bicarbonate (MT-25-035-CI, Corning) and 1 mM sodium pyruvate (MT-25-000-CI, Corning) at 37 °C, 5% CO_2_.

### 4.4. ePRP Treatments

Each vial of the ePRP powder was carefully reconstituted in 1 mL of growth medium under aseptic conditions and completely mixed by pipetting up and down to dissolve all of the powder. The designated number of fibroblasts was seeded on the plate for the experiment based on the assay requirements. The next day, cells were co-cultured in the conditioned medium for 24 h with varying doses of the ePRP solution at 37 °C, 5% CO_2_. After 24 h of treatment, the conditioned medium was removed and the cells were washed once with PBS (MT-21-040-CM, Corning). Then, the appropriate volume of the standard culture medium for cell growth was added.

### 4.5. Cell Proliferation Assay

5 × 10^3^ cells per well were seeded into a 96-well plate and cultured for 24 h in the presence or absence of 5%, 10%, 15%, 20%, 25%, 30%, 40%, and 50% ePRP at 37 °C, 5% CO_2_. After 48 and 72 h of post-growth culture, absorbance was measured at 450 nm (SpectraMax^®®^ M2e Microplate Reader, Molecular Devices LLC., San Jose, CA, USA) using CCK-8 reagent (Cat. CK04-20, Dojindo, Kumamoto, Japan). OD values were used to compare the viability of ePRP-treated and non-treated cells.

### 4.6. Wound Healing Migration Assay

5 × 10^4^ cells were seeded into ibidi culture inserts (Cat. 80209, ibidi GmbH, Munich, Germany) in a 24-well plate and allowed to grow overnight to evaluate how ePRP influenced fibroblast migration. The inserts were then removed, and the medium was replaced with normal media containing or not containing the different quantities of ePRP (5% and 10%). After 24 h of incubation, the conditioned medium was removed, and images of the wound scratch were taken at the moment of removal (0 h) and at regular intervals for a total of 48 or 72 h.

### 4.7. RNA Isolation and Real-Time RT-PCR

The GENEzol Pure Kit (Cat. GZX200, Geneaid Biotech Ltd., New Taipei City, Taiwan) was utilized to isolate the total RNA from the CCD-966SK cells. The purified RNA was kept at −80 °C after being eluted in 30 μL of nuclease-free water. The RNA concentration was assessed using a NanoDrop 2000 (Cat. ND2000CLAPTOP, Thermo Fisher Scientific), and the quality of the extracted RNA was in the range of 1.9–2.3 and 1.8–2.0, as determined by absorbance ratios of A260/A230 and A260/A280. The SensiFAST cDNA Synthesis Kit (Cat. BIO-65054, Bioline, Memphis, TN, USA) was employed to synthesize the cDNA according to the manufacturer’s instructions. To test the levels of the human metabolic gene, RT-PCR amplification was used. All primer sets were designed to precisely amplify a region encompassing exons of each gene (Table S1). The SensiFAST SYBR Hi-ROX Kit (BIO-92005, Bioline) was utilized, and the amplification was detected using a StepOnePlus Real-Time PCR Systems (Thermo Fisher Scientific). The 2^−ΔΔCt^ method was used to determine relative gene expression, with data normalized to the EEF1A1 housekeeping gene.

### 4.8. Measurement of the Intracellular ATP Content

5 × 10^3^ cells per well were seeded into a white 96-well plate and treated with 5% and 10% ePRP for 24 h at 37 °C, 5% CO_2_. After 24 and 48 h of post-growth culture, the intracellular ATP level was determined using the Luminescent ATP Detection Assay Kit (Cat. ab113849, Abcam, Cambridge, UK) according to the manufacturer’s protocol. The intensity of fluorescence in comparison to the non-treated cells was used to calculate the relative intracellular ATP level.

### 4.9. Flow Cytometry Analysis

For flow cytometry analysis, an Attune NxT Flow Cytometer (Thermo Fisher Scientific) and FlowJo software (Tree Star, Ashland, OR, USA) were used. Following the ePRP treatment, 1 × 10^6^ fibroblasts were cultured for 15, 30, and 60 min at 37 °C in normal MEM with 5 μM CellROX green (Cat. C10444, Thermo Fisher Scientific) and 2.5 μM NAO (Cat. A1372, Thermo Fisher Scientific) in normal MEM. After staining, the cells were washed 3 times with a buffer (PBS pH 7.2, 0.5% bovine serum albumin) and centrifuged for 5 min at 1500× *g*. After gating viable cells by forward scatter (FSC) and side scatter (SSC) signal, 1 × 10^4^ events were collected for each sample for flow cytometry analysis.

### 4.10. Senescence β-Galactosidase Staining

Adherent cells were seeded into the 6-well plate at a density of 1 × 10^5^ cells per well. At 48 and 72 h following the 5% and 10% ePRP treatments, the cells were rinsed in PBS and fixed by a fixation solution for 15 min at room temperature before being stained at least overnight in a non-CO_2_ incubator according to the manufacturer’s instructions. The ratio of β-galactosidase-positive cells was calculated using ImageJ software (National Institutes of Health, Bethesda, MD, USA).

### 4.11. Metabolic Flux Assays

The ePRP-treated cells were seeded in the Seahorse XF96 Cell Culture Microplate (Cat. 102340-100, XFe24 FluxPaks, Agilent, Santa Clara, CA, USA) on the day before the experiment at 1.2 × 10^4^ cells per well in the standard culture medium. The utility plate (Cat. 102340-100, XFe24 FluxPaks, Agilent) was filled with 1 mL Seahorse XF Calibrant (Cat. 100840-000, Agilent), then incubated overnight in a non-CO_2_, 37 °C incubator. The cell culture medium should be replaced with Seahorse assay medium (Cat. 103575-100, DMEM, Agilent) the next day. XF Cell Stress Test (Cat. 103015-100, Agilent) chemicals were used for the experiment according to the manufacturer’s instructions. The cell culture medium should be replenished with Seahorse assay medium (Cat. 103575-100, DMEM, Agilent) the very next day. XF Cell Stress Test (Cat. 103015-100, Agilent) compounds were used to perform the experiment according to the manufacturer’s instructions. The Seahorse XFe24 Analyzer (Agilent) was used to quantify metabolic flux. The rate of extracellular acidification (ECAR) denotes the amount of lactate generated by cells as a result of glycolysis, whereas the rate of oxygen consumption (OCR) represents the rate of mitochondrial respiration.

### 4.12. Statistical Analysis

Statistical analyses were performed using Graphpad Prism 8, and *p*-values of 0.05 or less were considered significant. The data were presented as the mean ± SEM of independent experiments. Depending on the type of data, several statistical tests such as the Student’s t-test, one-way ANOVA, and two-way ANOVA have been used. Statistically significant differences are shown by the symbols * *p* < 0.05, ** *p* < 0.01, *** *p* < 0.001, and **** *p* < 0.0001.

## Data Availability

The data presented in this study are available on request from the corresponding author.

## Supplementary Materials

The following are available online at https://www.mdpi.com/article/10.3390/ijms222312623/s1.
