# Supplementary material for: Enhanced Platelet-Rich Plasma (ePRP) Stimulates Wound Healing through Effects on Metabolic Reprogramming in Fibroblasts
Source: Int J Mol Sci. 2021 Nov 23;22(23):12623. doi: 10.3390/ijms222312623 (PMC8657780; doi:10.3390/ijms222312623)
Supplement: Supplementary file 1 [file ijms-22-12623-s001.zip › ijms-1466730-supplementary.pdf]

Supplemental Tables

Table S1. List of real-time PCR (RT-qPCR) primers

Gene name	NCBI RefSeq	Primer Sequence	Product size (bp)
HK2	NM_001371525.1	F :CCCTGCCACCAGACTAAACT<br>R :TGGACTTGAATCCCTTGGTC	64
GPI	NM_001329911.2	F :TCTATGCTCCCTCTGTGTTAGA<br>R :CTCCTCCGTGGCATCTTTATT	114
PFKM	NM_001354742.2	F :AGGAGGGGAAGGGCATCT<br>R :TTCCTATCAAATGGGGTTGG	85
GAPDH	NM_001357943.2	F :GGTGTGAACCATGAGAAGTATGA<br>R :GAGTCCTTCCACGATACCAAAG	123
LDHA	NM_001165416.2	F :AGATTCCAGTGTGCCTGTATG<br>R :ACCTCTTTCCACTGTTCCTTATC	108
PDK	NM_002610.5	F :ACGCTGGGTAATGAGGATTTG<br>R :GAGGTCTTGGTGCAGTTGAATA	112
PDHA1	NM_001173455.2	F :AGGTTTGCTGCTGCCTATT<br>R :ACTCCAGGGTCACTCATACT	98
POLG1	NM_002693.3	F :GAGTTCCTGCTCACTGACAATAG<br>R :GCTCGCAAGTTCTCCATCTT	98
POLG2	NM_007215.4	F :GCTCGCAAGTTCTCCATCTT<br>R :ATGCTGGCCTACCTCTATGA	103
TFAM	NM_001270782.2	F :ATGGCGTTTCTCCGAAGCAT<br>R :CAGATGAAAACCACCTCGGTAA	133
PGC-1α	NM_001330751.2	F :AGAGCGCCGTGTGATTTAT<br>R :CTCCATCATCCCGCAGATTTA	122
PGC-1β	NM_133263.4	F :CCACATCCTACCCAACATCAA<br>R :GTGCTGTCCGCCTTAACA	109
NRF1	NM_005011.5	F :GCAAGCTATTGTCCTCTGTATCT<br>R :ACTTACGCACCACATTCTCC	92
OGG1	NM_016829.3	F :ATCGTACTCTAGCCTCCACTC<br>R :ACCGGAAAGATTGTCCAGAAG	102
Cu/ZnSOD	NM_000454.5	F :CTCAGGAGACCATTGCATCAT<br>R :TTCCAGCGTTTCCTGTCTTT	107
MnSOD	NM_001322819.2	F : GGGTTGGCTTGGTTTCAATAAG	120

		R :TGCTCCCACACATCAATCC	
Catalase	NM_001752.4	F :CTGGAGCACAGCATCCAATA<br>R :TCATTCAGCACGTTCACATAGA	104
GPX1	NM_001329455.2	F :ACGCCAAGAACGAAGAGATT<br>R :CGTTCACCTCGCACTTCT	99
GPX4	NM_001367832.1	F :GGGAGTAACGAAGAGATCAAAGAG<br>R :GGATCTTCATCCACTTCCACAG	118
GSR	NM_001195102.3	F :CGGTGCCAGCTTAGGAATAA<br>R :GCCATCTCCACAGCAATGTA	102
GSS	NM_000178.4	F :GATGGACTTCAACCTGCTAGTG<br>R :GTCAAAGAGACGAGCGGTAAA	109
EEF1A1	NM_001402.6	F :GCTGGAAGATGGCCCTAAAT<br>R :CCAAAGGTGGATAGTCTGAGAAG	107
SIRT1	NM_001142498.2	F : AGTGGCAAAGGAGCAGATTAG<br>R :CTGCCACAAGAACTAGAGGATAAG	138
